# Pedro Pons’ sign of brucellar spondylitis

**DOI:** 10.1590/0037-8682-0561-2019

**Published:** 2020-03-16

**Authors:** Na Wu, Yi Zhang, Yong-Sheng Yu

**Affiliations:** 1 Department of Infectious Diseases, Shanghai Jiao Tong University Affiliated Sixth People’s Hospital, Shanghai, China.

**FIGURE 1:**
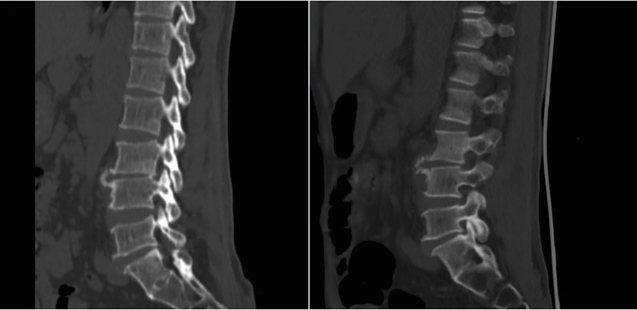
Computed tomography scan showing apparent destruction at the anterior superior corner of the L4 vertebra (Pedro Pons’ sign) accompanied by prominent osteosclerosis and osteophytes resembling a parrot’s beak.

**FIGURE 2:**
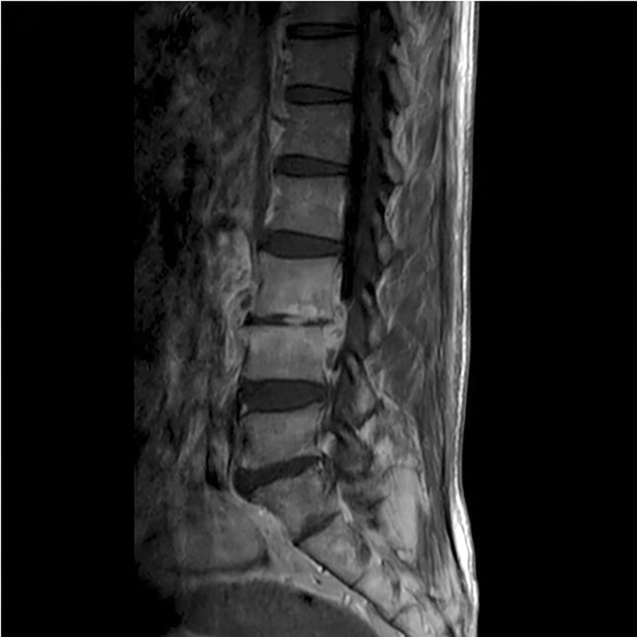
Magnetic resonance imaging showing bone destruction in the L3 and L4 vertebrae, narrowed intervertebral space, and paravertebral abscesses resembling teardrops.

A 62-year-old man in close contact with sheep presented with a one-month history of intermittent fever, fatigue, night sweats, and progressive low back pain. Physical examination revealed percussion pain in the L3 and L4 vertebrae. Laboratory data showed a white blood cell count of 5.6 × 10^9^/L with 59.5% neutrophils and 27.8% lymphocytes. Inflammatory marker levels were elevated with erythrocyte sedimentation rate of 107 mm/h and C-reactive protein of 65 mg/L. A computed tomography (CT) scan revealed apparent destruction at the anterior superior corner of the L4 vertebra, known as Pedro Pons’ sign, accompanied by osteophyte formation (Figure 1). Magnetic resonance imaging showed bone destruction in the L3 and L4 vertebrae accompanied by paravertebral abscesses (Figure 2). The patient underwent CT-guided percutaneous catheter drainage. *Brucella ovis* (*B. ovis*) was isolated from the pus. Furthermore, the serum agglutination test was positive for *Brucella* at a titer of 1:800. With a diagnosis of brucellar spondylitis, the patient received three months of antibiotic therapy and had a satisfactory response to the medical treatment.

Pedro Pons’ sign was first described by Pedro-Pons and Farreras in 1944 as a destructive appearance at the anterosuperior angle of the vertebra accompanied by osteosclerosis and osteophytes, which are characteristic radiological findings of brucellar spondylitis^1^ ^,^ ^2^.
